# Acknowledgement to reviewers

**DOI:** 10.1111/anec.12738

**Published:** 2020-01-09

**We are grateful to the 2019 Reviewers:**

Mehmeh Aktas

Jeffrey Alexis

Mohamed Faher Almahmoud

Jesus Almendral

Wim Anné

Aapo Aro

Ljuba Bacharova

Fabio Badilini

Adrian Baranchuk

Riccardo Barbieri

Axel Bauer

Antonio Bayes de Luna

Lennart Bergfeldt

Yochai Birnbaum

David Blehar

Konrad Brockmeier

Yong‐Mei Cha

Shih‐Ann Chen

Yundai Chen

Keping Chen

Lin Y. Chen

Lovely Chhabra

Erin Coakley

Daniel Cortez

Jean‐Philippe Couderc

Bettina Cuneo

Iwona Cygankiewicz

Borje Darpo

James Daubert

Polychronis Dilaveris

Ward C. Dobbs

Sergio Dubner

Nabil El Sherif

Martha Felini

Miquel Fiol

Javier García‐Niebla

Sandro Gelsomino

Ilias Giallafos

Ilan Goldenberg

Anton Gorgels

Mark Haigney

Arto Hautala

Tom Heinink

Fredrik Holmqvist

David Huang

Salim Idriss

Takanori Ikeda

Junichi Ishi

Atila Iyisoy

Lars Johannesen

Christian Jons

Demosthenes G Katritsis

Michal Katz‐Leurer

Helmut Klein

Paul Kligfield

Thomas Klingenheben

Tetsuo Konno

Rungroj Krittayaphong

Piotr Kukla

Valentina Kutyifa

Maria Teresa La Rovere

Gabriel Laurent

Anthony Leicht

Emanuela Locati

Peter Macfarlane

John Madias

Rahhael Martins

Jay Mason

Craig McPherson

Naoki Misumida

Suneet Mittal

Hiroshi Morita

David Mortara

Saman Nazarian

Tamim Nazif

Kjell Nikus

Antonio Claudio Lucas da Nobrega

Tomas Novotny

Jai‐Wun Park

Rod Passman

Tiago Peçanha

Juha Perkiomaki

Filip Van Petegem

Ryszard Piotrowicz

Ewa Piotrowicz

Pyotr Platonov

Mark Potse

Frits W. Prinzen

Pawel Ptaszynski

Helmut Puererfellner

Jennifer Reed

Spencer Rosero

Jerzy Sacha

Tapio SeppAnen

Karan Singh

Peter Smars

Elsayed Soliman

Phyllis Stein

Jonathan Steinberg

Martin Stockburger

Peter Stone

David Strauss

Cees A. Swenne

Masahiko Takagi

Bonpei Takase

Larisa Tereshchenko

Svjetlana Tisma‐Dupanovic

Maria Trusz‐Gluza

Gary Tse

Georg Van Hare

Richard Verrier

Ronald Wakai

Stafford Warren

Stefan Weber

Arthur Wilde

Jerzy Wranicz

Koichiro Yoshioka

Grazyna Zareba

Karolina Zareba

Wojciech Zareba

